# Supplementary material for: Correlative Microscopy of Vitreous Sections Provides Insights into BAR-Domain Organization In Situ
Source: Structure. 2018 Jun 5;26(6):879–886.e3. doi: 10.1016/j.str.2018.03.015 (PMC5992340; doi:10.1016/j.str.2018.03.015)
Supplement: Document S1. Figures S1–S3 [file mmc1.pdf]

Structure, Volume *26*

Supplemental Information

Correlative Microscopy of Vitreous Sections Provides Insights into BAR-Domain Organization *In Situ*

Tanmay A.M. Bharat, Patrick C. Hoffmann, and Wanda Kukulski

Supplemental Figure S1

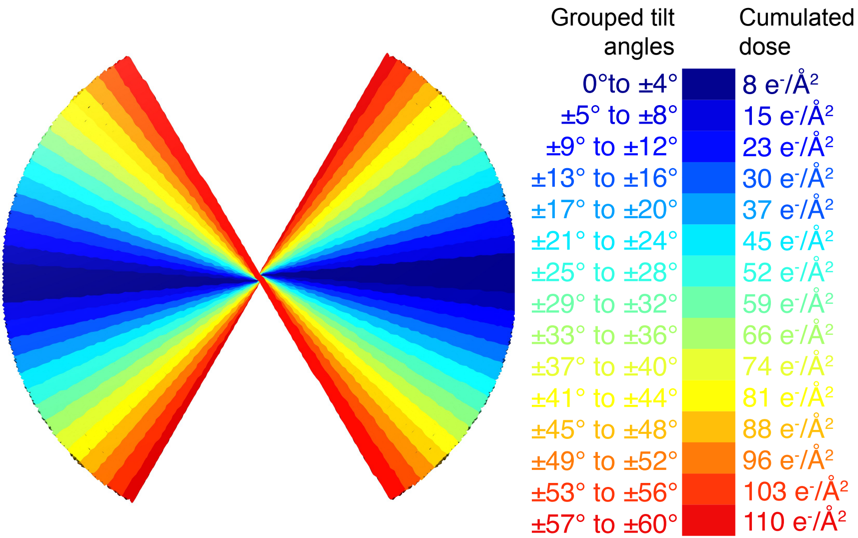

Supplemental Figure S2

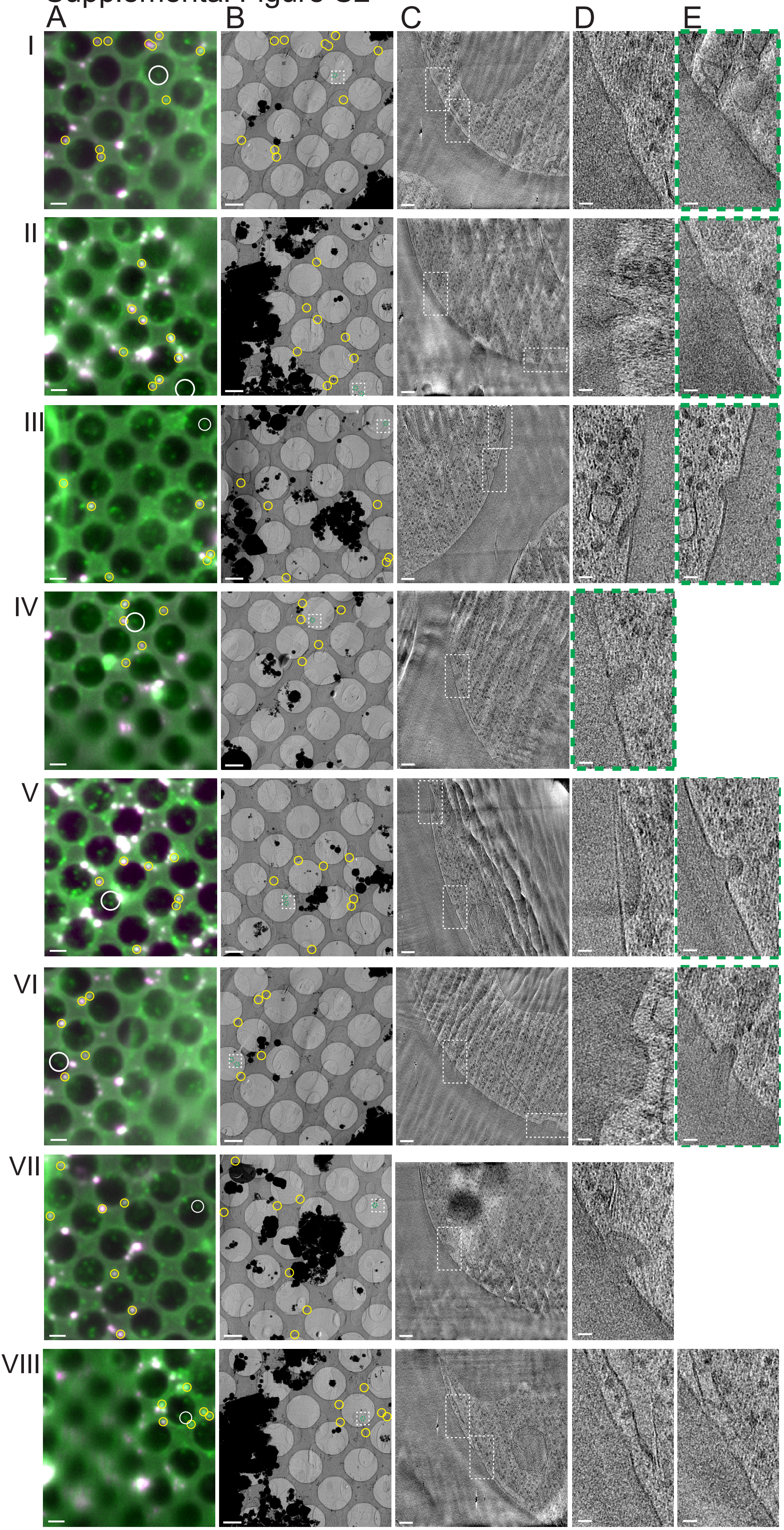

Supplemental Figure S3

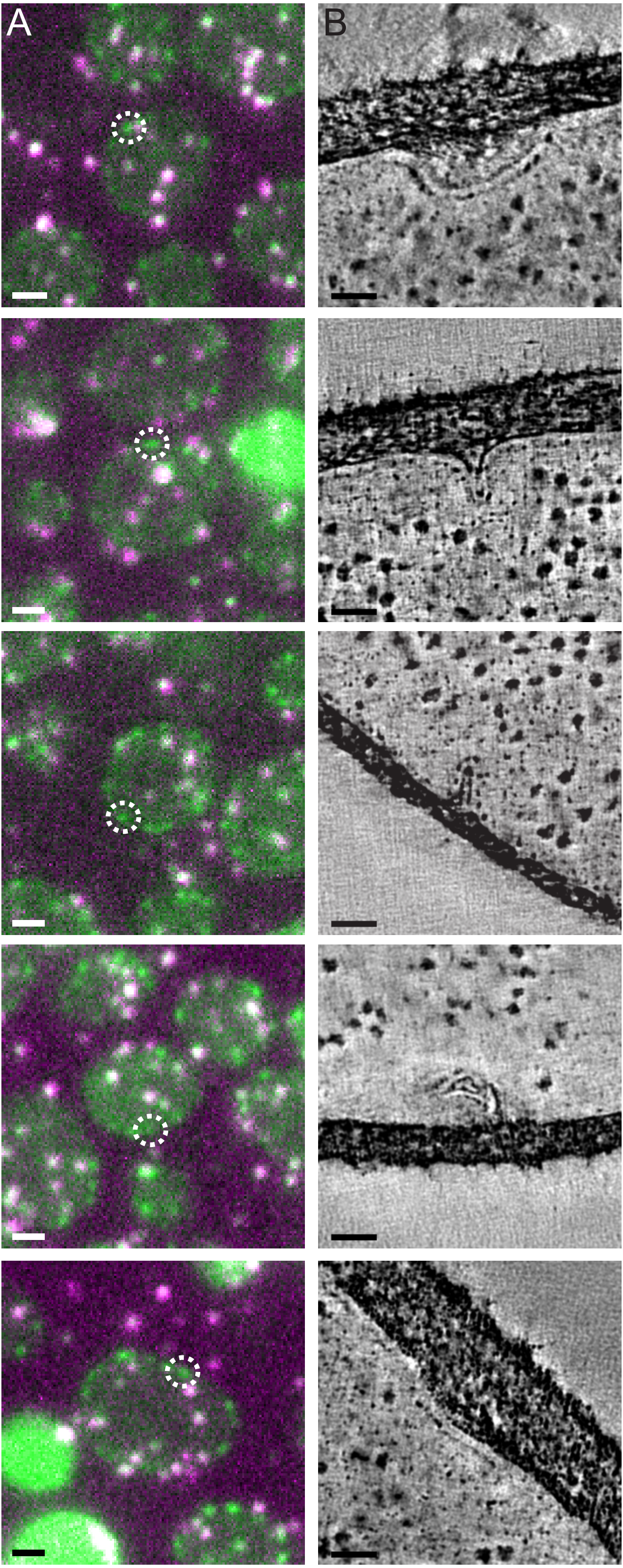

Supplemental Figure Legends:

Supplemental Figure S1: Tilt scheme. Related to Figure 2. We applied a grouped dose-symmetric tilt scheme for cryo-ET data acquisition, where the goniometer is successively tilted in opposite directions (Hagen et al., 2017). Four images were collected in succession in one direction (relative to the untilted 0° image), followed by four images in the other direction, while moving from 0° to ±60°. Changing direction after every four images proved to be time-efficient, as well as dose-efficient, since high cumulated doses were restricted to high tilt images. This allowed discarding high tilt images without compromising the lower tilt images.

Supplemental Figure S2: Gallery of cryo-correlative microscopy and corresponding cryo-ET data. Related to Figures 2 and 3. A: Single focal plane cryo-FM images, merge of red (shown in magenta) and green channels, rotated, cropped and scaled to match the corresponding intermediate magnification cryo-EM images shown in **B**. The coordinates of the Pil1-GFP signals indicated by the white circle (A), were transformed using the fiducial markers highlighted by the yellow circles in A and B. The centres of the green dashed circles (B) are the predicted positions of Pil1-GFP signals of interest. Note that the white circles (A) in rows II, V and VI contain two Pil1-GFP signals each. The white dashed rectangles correspond to the field of view imaged by cryo-ET, shown in **C** as a single virtual slice through the reconstructed tomogram. Dashed rectangles in C indicate the location of the eisosomes shown in **D** and **E**. The images in D and E are magnifications of the virtual slice shown in C, except for EI and EII, which are magnifications of different virtual slices than those shown in C. Green dashed boxes indicate eisosomes that are also shown in Figure 3 and that have resulted in interpretable 2D class averages of eisosomal as well as nearby plasma membrane. Due to a defect of the direct electron detector, the EM images have a stripe of faulty pixels. They appear as a narrow stripe of repeating image features apparent in some of the images shown in B. Images shown in

EI and Figure 3BI, EII and Figure 3BII, DIV and Figure 3BIV, and EV and Figure 3BV, are details from the same image, respectively. Scale bars: 2 *μ*m in A and B, 100 nm in C and 25 nm in D and E.

Supplemental Figure S3: Room-temperature correlative light and electron microscopy of Pil1-GFP. Related to Figure 3. Room-temperature correlative light and electron microscopy confirms that eisosomes correspond to furrow-like invaginations that are oriented at various angles relative to the plane of the plasma membrane, as well as shallow indentations of the plasma membrane. **A:** Room-temperature fluorescence microscopy of 300 nm sections of yeast cells embedded in resin, on EM grids. Overlay of green (Pil1-GFP) and far-red (fiducial markers, shown in magenta) signals. **B:** Virtual slices through scanning transmission electron microscopy tomograms acquired at the predicted positions of the Pil1-GFP spots marked by white dashed circles in A. Scale bars: 1 *μ*m in A, 50 nm in B.
