# Supplementary material for: Correlative Microscopy of Vitreous Sections Provides Insights into BAR-Domain Organization In Situ
Source: Structure. 2018 Jun 5;26(6):879–886.e3. doi: 10.1016/j.str.2018.03.015 (PMC5992340; doi:10.1016/j.str.2018.03.015)
Supplement: Document S2. Article plus Supplemental Information [file mmc2.pdf]

Short Article

Structure

Correlative Microscopy of Vitreous Sections Provides Insights into BAR-Domain Organization *In Situ*

Graphical Abstract

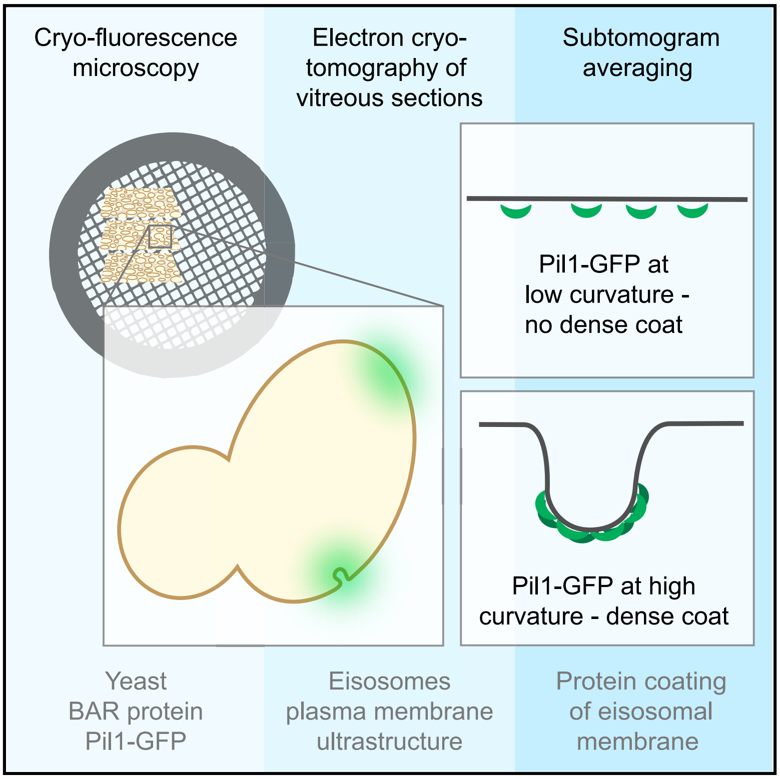

Authors

Tanmay A.M. Bharat,
Patrick C. Hoffmann, Wanda Kukulski

Correspondence

kukulski@mrc-lmb.cam.ac.uk

In Brief

Macromolecular assemblies can be visualized in their cellular environment by electron cryo-tomography. However, data acquisition and identification of elusive structures are major bottlenecks hindering widespread application. Bharat et al. address these issues by cryo-correlative microscopy, and use their workflow to study the formation of BAR-domain coats on cellular membranes.

Highlights

- Cryo-fluorescence microscopy eases electron cryo-tomography of vitreous sections
- Elusive protein assemblies are localized *in situ* by correlative microscopy
- Yeast BAR-domain protein Pil1 binds to plasma membrane with varying curvature
- Scaffold-like coats are only seen when Pil1 is bound to high curvature membranes

Bharat et al., 2018, Structure *26*, 879–886
June 5, 2018 © 2018 MRC Laboratory of Molecular Biology.
Published by Elsevier Ltd.
https://doi.org/10.1016/j.str.2018.03.015

Structure
Short Article

Correlative Microscopy of Vitreous Sections Provides Insights into BAR-Domain Organization *In Situ*

Tanmay A.M. Bharat,[1,2,3] Patrick C. Hoffmann,[4] and Wanda Kukulski[4,5,*]
[1]Sir William Dunn School of Pathology, University of Oxford, South Parks Road, Oxford OX1 3RE, UK
[2]Central Oxford Structural and Molecular Imaging Centre, South Parks Road, Oxford OX1 3RE, UK
[3]Structural Studies Division, MRC Laboratory of Molecular Biology, Francis Crick Avenue, Cambridge CB2 0QH, UK
[4]Cell Biology Division, MRC Laboratory of Molecular Biology, Francis Crick Avenue, Cambridge CB2 0QH, UK
[5]Lead Contact
*Correspondence: kukulski@mrc-lmb.cam.ac.uk
https://doi.org/10.1016/j.str.2018.03.015

SUMMARY

Electron microscopy imaging of macromolecular complexes in their native cellular context is limited by the inherent difficulty to acquire high-resolution tomographic data from thick cells and to specifically identify elusive structures within crowded cellular environments. Here, we combined cryo-fluorescence microscopy with electron cryo-tomography of vitreous sections into a coherent correlative microscopy workflow, ideal for detection and structural analysis of elusive protein assemblies *in situ*. We used this workflow to address an open question on BAR-domain coating of yeast plasma membrane compartments known as eisosomes. BAR domains can sense or induce membrane curvature, and form scaffold-like membrane coats *in vitro*. Our results demonstrate that in cells, the BAR protein Pil1 localizes to eisosomes of varying membrane curvature. Subtomogram analysis revealed a dense protein coat on curved eisosomes, which was not present on shallow eisosomes, indicating that while BAR domains can assemble at shallow membranes *in vivo*, scaffold formation is tightly coupled to curvature generation.

INTRODUCTION

Biological macromolecules fulfill their tasks in crowded cellular environments. The organization of macromolecular functions in the cell often necessitates formation of transient complexes, such as proteins binding to membranes or to the cytoskeleton, or the self-association of proteins into oligomers. Such interactions can generate short-lived or fragile higher-order assemblies, whose formation can depend on the local cellular environment, for example the composition of the organelle membrane at which they occur. Understanding how macromolecular assemblies contribute to cellular function is a long-term goal of many structural and cell biological endeavors. The essential steps toward this goal are identifying, visualizing, and solving structures of macromolecular assemblies in their native environment.

Advances in electron cryo-tomography (cryo-ET) have resulted in improved visualization of protein assemblies inside cells (Beck and Baumeister, 2016). However, efficient data acquisition from cellular samples and identification of structures within cells remain two major bottlenecks for routine cellular structural biology applications. Firstly, due to limitations imposed by the electron beam, unless thin areas of cells (<0.5 μm), such as the cell cortex, are being imaged, physical thinning of specimens is required either by vitreous sectioning or using focused ion beam (FIB)-milling (Al-Amoudi et al., 2007; Marko et al., 2007). Both vitreous sectioning and FIB-milling are cumbersome methods in terms of generating samples of sufficiently high quality for subsequent tilt series data acquisition. Secondly, in the acquired cryo-ET data, identification of features of interest remains a problem. While large protein complexes, such as ribosomes or proteasomes, can be readily localized within the tomographic cell volume using template-matching algorithms (Asano et al., 2015; Pfeffer et al., 2015), smaller protein assemblies that lack such large characteristic shapes remain elusive.

In this study, we have addressed both these limitations by implementing a high-precision correlative microscopy workflow that combines cryo-fluorescence microscopy (cryo-FM) with cryo-ET of vitreous sections. Our approach provides certainty that the area to be imaged in the electron microscope (EM) contains the structure of interest and is of sufficient quality for cryo-ET data acquisition. Moreover, our procedure facilitates localization of elusive protein assemblies within the acquired cryo-ET volume.

We have applied this workflow to answer a long-standing question on the assembly of BAR (Bin/amphiphysin/Rvs) domain-containing proteins by visualizing them directly on cellular membranes. BAR domains are alpha-helical bundles that bind to membranes and can induce or sense various membrane curvatures according to their shape (McMahon and Gallop, 2005). For instance, BAR proteins assist in membrane remodeling during vesicle formation (Gallop et al., 2006; Kukulski et al., 2012; Peter et al., 2004). *In vitro*, these proteins efficiently tubulate liposomes (Peter et al., 2004; Youn et al., 2010), and can assemble into regular lattices that have been characterized structurally, revealing the arrangement of BAR domains on lipid

This is an open access article under the CC BY license (http://creativecommons.org/licenses/by/4.0/).

tubules (Daum et al., 2016; Frost et al., 2008; Mim et al., 2012). The yeast BAR proteins Lsp1 and Pil1 are highly similar to human amphiphysin and endophilin (Ziółkowska et al., 2011), and are the main constituents of plasma membrane compartments called eisosomes (Olivera-Couto et al., 2015; Walther et al., 2006). Eisosomes form stable membrane furrows (Strádalová et al., 2009), involved in sphingolipid metabolism mediated by TOR Complex 2, maintenance of the cellular phosphoinositide pool and cell polarity (Berchtold et al., 2012; Fröhlich et al., 2014; Seger et al., 2011). The regular lattice formed by Lsp1 and Pil1 *in vitro* suggests a model for scaffolding of the furrows (Karotki et al., 2011), supported by the high density of membrane-bound Pil1 observed in live cells (Lacy et al., 2017). Interestingly, flattening of eisosomes has been proposed as a mechanism for membrane expansion (Kabeche et al., 2015), indicating that protein assemblies on eisosome membranes may undergo changes depending on subcellular conditions.

Thus, how the BAR-domain organizations observed in minimal reconstituted systems relate to the more complex and varying environment of cellular membranes remains to be seen. Presumably, local membrane tension, the density of BAR domains, and the presence of other curvature-generating proteins can impact scaffold formation by BAR proteins, as well as their ability to induce curvature (Simunovic et al., 2015).

We found our correlative microscopy workflow ideal to study how membrane curvature generation and BAR protein assembly relate to each other in cells. Our *in situ* cryo-ET data reveals that the organization of proteins on eisosomal membranes varies with membrane curvature. These results demonstrate how our correlative workflow can be used to obtain targeted, high-resolution cryo-ET data on elusive protein assemblies within thick regions of cells, supporting *in situ* structural and cell biology studies.

RESULTS

Cryo-FM Facilitates Efficient Cryo-ET Acquisition on Vitreous Sections

Our first goal was to unambiguously identify and visualize a set of eisosomes representative of their ultrastructural diversity using cryo-ET. Therefore, we prepared thinned yeast cells expressing Pil1-GFP by vitreous sectioning (CEMOVIS) of high-pressure frozen yeast pellets (Al-Amoudi et al., 2004). Serial vitreous sections form ribbons that comprise very large areas of thinned specimen. Given the small size of yeast cells, and their density in the cell pellet, each EM grid can contain thousands of cell profiles and, thus, the number of eisosomes accessible for cryo-ET imaging is large. Moreover, fiducial-based correlation using cryo-FM (Schorb et al., 2016) can be adapted to vitreous sections to unambiguously locate eisosomes with high spatial precision.

We used a recently developed cryo-FM system that combines an upright wide-field microscope with a liquid nitrogen-cooled stage (Schorb et al., 2016). First we acquired a tile scan of the whole grid in the green channel (Figure 1A). This allowed us to evaluate the overall quality of the grid and locate 5–10 grid squares that contained intact carbon film covered with vitreous sections. We manually moved to each of these grid squares and acquired focal stacks in green and red channels (Figures 1B and 1C). The red channel images were acquired to distinguish fiducial markers, which fluoresce in both channels, from the Pil1-GFP signal.

We found that the cryo-FM focal stacks were essential downstream for efficient acquisition of cryo-ET data from vitreous sections. Cryo-ET studies on vitreous sections have been considered difficult because of unevenness and incomplete attachment of the sections to the carbon support film (Bouchet-Marquis and Hoenger, 2011; Hsieh et al., 2006). These imperfections cause specimen movement during data acquisition, resulting in blurred images and tilt series that are difficult or impossible to align (Al-Amoudi and Frangakis, 2013; Pierson et al., 2010; Sartori Blanc et al., 1998). In our focal stacks, we inspected whether the Pil1-GFP signals appeared in the same focal plane as the signals of fiducial markers or the background fluorescence of the carbon film. Areas in which all signals were in focus in the same focal plane were likely to be flat and well attached (Figures 1B and 1E). In areas where this was not the case, the section was located several micrometers away from the plane of the carbon film, indicating that it was not well attached (Figures 1C and 1G). Within the areas of flat, attached sections, we further preselected carbon film holes that contained Pil1-GFP signals for acquisition of tomograms (Figure 1E).

After cryo-FM imaging, the grid was transferred to the EM, where we acquired montaged images of the preselected grid squares at an intermediate magnification to find cell profiles containing the preselected Pil1-GFP spots (Figure 1D). Based on visual correlation between cryo-FM images and the intermediate-magnification EM maps, we marked the positions for cryo-ET data collection where we expected eisosomes. Orientation was eased by two types of visual cues that were readily recognizable in the maps: the plasma membrane at which eisosomes are located, and the TetraSpeck fiducial markers adhered to the carbon film, which we could assign to signals in the fluorescence image (Figures 1D and 1E and STAR Methods).

Notably, in the intermediate-magnification EM maps, the areas that were identified by cryo-FM as not being well attached, appeared indistinguishable from those identified as well attached (Figures 1D and 1F). In comparison with FM, the depth of field in EM is large, which is why differences in height between section and carbon film are easier to assess by FM. We found that in all cases the areas we preselected were stable and allowed us to collect cryo-ET data using a dose-symmetric tilt acquisition scheme (Figure S1) (Hagen et al., 2017), indicating that our assessment and preselection by cryo-FM was efficient.

BAR-Domain Protein Pil1 Localizes to Membrane Furrows and to Flat Membranes

To unambiguously localize Pil1-GFP within the cell segments imaged using cryo-ET, we computed precise transformations of the GFP spot coordinates into the intermediate-magnification maps using the TetraSpeck fiducial markers, visible both in the cryo-FM images (Figures 2A and S2A) and in the EM maps (Figures 2B and S2B) (Kukulski et al., 2011; Schorb et al., 2016). At the corresponding positions in the electron tomograms, the majority of locations (9 out of 12 localized Pil1-GFP signals) showed invaginations of the plasma membrane (Figures 2C, 2D, 3A, 3B, and S2). One of the Pil1-GFP localizations revealed two invaginations (Figure S2III). The appearance of the total of

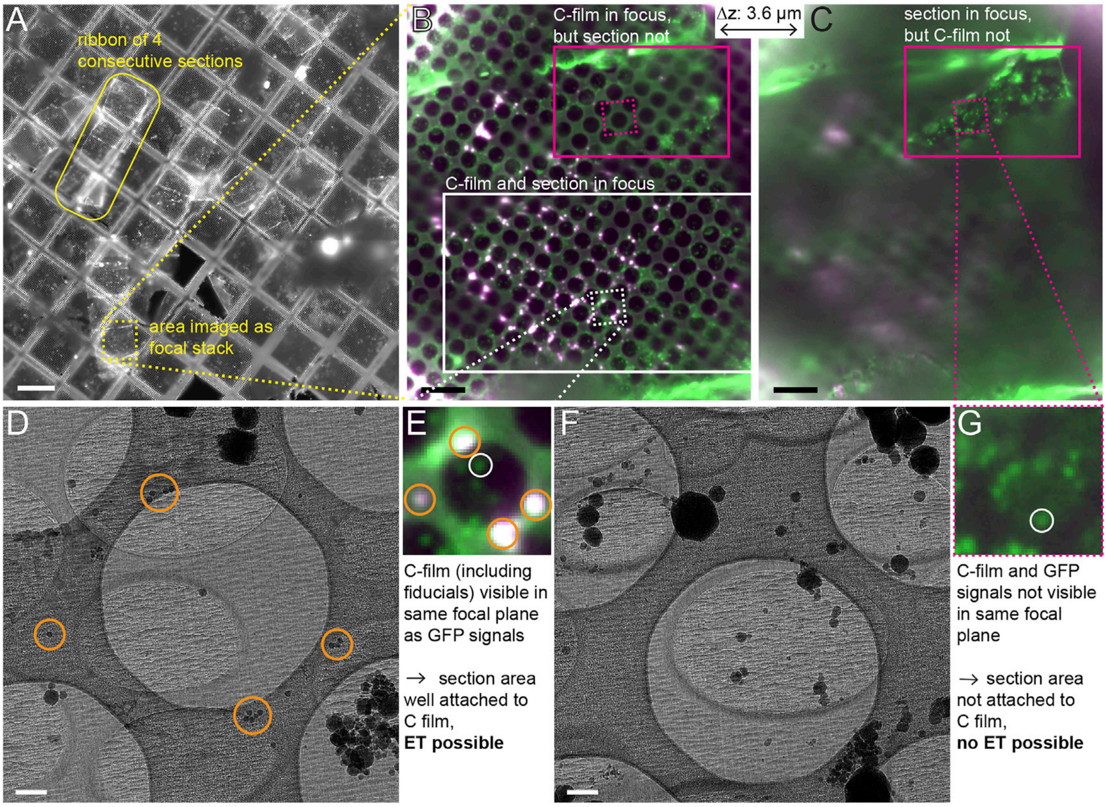

Figure 1. Cryo-FM Allows Selection of Vitreous Section Areas that Are Suitable for Cryo-ET and Contain Fluorescent Signals of Interest

(A) Grid overview in the green channel shows the distribution of vitreous sections appended to each other as ribbons.

(B and C) Two planes from the same focal stack, separated by about 3.6 μm in z (direction of the light path), merge of red (shown in magenta) and green channels. Area of acquisition corresponds to the yellow dashed box in (A). The white box in (B) indicates a section area where the holey carbon film and the GFP signals in the section are in focus in the same plane. The magenta box indicates a section area where the carbon film is in focus in the plane shown in (B), whereas the GFP signals are in focus in a different plane shown in (C).

(D) Intermediate-magnification EM image of the area indicated by the white dashed square in (B), magnified in (E).

(E) The white circle marks a Pil1-GFP signal suitable for cryo-ET acquisition. The orange circles in (D and E) mark examples of fiducial markers.

(F) Intermediate-magnification EM image of the area indicated by the magenta dashed square in (B and C), magnified in (G).

(G) The white circle indicates a Pil1-GFP spot not suitable for cryo-ET acquisition.

Scale bars represent 100 μm in (A), 10 μm in (B and C), 500 nm in (D and F).

ten invaginations varied in cross-section, which we attributed to differences in their orientation relative to the section plane, as well as to differences in depth, width, and overall shape. Eight of the invaginations appeared furrow-like, similar to those described before (Karotki et al., 2011; Strádalová et al., 2009). Two of the furrows were highly tilted relative to the plasma membrane (Figures S2DIII and S2DVII). In 3 of the 12 identified Pil1-GFP positions, there was no furrow in the plasma membrane. Instead, the plasma membrane displayed shallow indentations, indicating that Pil1 was bound to a flat or weakly deformed plasma membrane (Figures 3BI, 3BII, S2I, S2II, and S2VIII). To verify that the orientation of the tilted furrows was not due to artifacts of vitreous sectioning, we imaged eisosomes using correlative microscopy of resin-embedded cells (Kukulski et al., 2011). In the resulting electron tomograms, Pil1-GFP localized to furrows tilted relative to the plasma membrane at various angles, as well as to shallow indentations of the plasma membrane, confirming that these eisosome shapes occur in yeast cells independent of the preparation method (Figure S3). In summary, applying a fiducial-based correlation procedure to vitreous sections allowed us to localize Pil1-GFP to membrane furrows of variable depth and shape, including very shallow indentations or flat regions of the plasma membrane.

The Protein Coat on Eisosomal Membranes Depends on Membrane Curvature

Many of the eisosomes, but not all, displayed a thicker layer of density as compared with the surrounding plasma membrane, which we attributed to the presence of a protein coat on the eisosome membrane. In particular, the highly curved ridges of furrows appeared to be coated (Figures 3BIII–3BVI). Regardless of whether or not the coat was discernable in the raw tomograms, we extracted overlapping subvolumes, centered along the eisosomal membrane, for sub-tomogram analysis (Figure 3B). Each subvolume (sub-tomogram) was collapsed into a 2D image (Bharat and Scheres, 2016), and the sets of 2D images from each eisosome were aligned, classified, and averaged using a regularized likelihood algorithm (Bharat et al., 2015). In

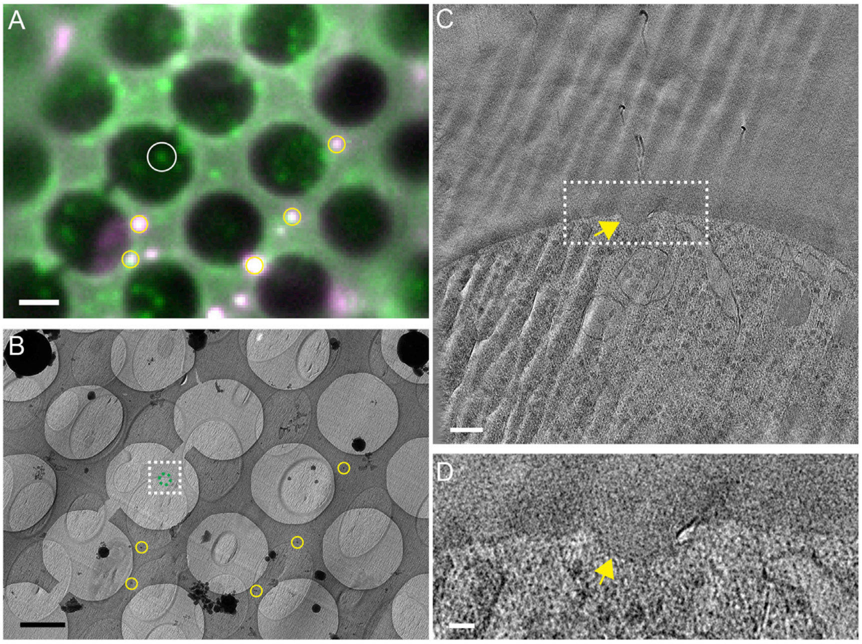

Figure 2. Cryo-ET of Preselected Areas and Fiducial-Based Correlation to Identify Eisosomes by Pil1-GFP Localization

(A) A single focal plane in cryo-FM, merge of green and red (shown in magenta) channels. White circle indicates a Pil1-GFP signal selected for cryo-ET.

(B) Intermediate-magnification EM image. White dashed rectangle indicates the field of view imaged by cryo-ET (C). Yellow circles in (A and B) indicate fiducial markers for high-precision localization of the Pil1-GFP signal (center of green dashed circle).

(C) Virtual slice through the tomographic volume.

(D) Magnified view of the dashed rectangle shown in (C). Arrows in (C and D) indicate a furrow-like invagination.

Scale bars represent 2 μm in (A and B), 100 nm in (C), and 25 nm in (D). See also Figure S2.

this way, we obtained 2D class averages of each eisosomal membrane, which allowed us to better assess the coating on every individual eisosome (Figure 3C). To ensure that our interpretation of the class averages would reflect the eisosome-specific structure, rather than be biased by cryo-ET imaging effects, such as defocus, for comparison we also generated class averages from patches of plasma membrane (Figure 3D), which were ~200 nm away from the eisosome (Figure 3B). For some eisosomes and plasma membrane patches, the sub-tomogram alignment did not yield meaningful 2D class averages. This was not surprising, as in many cases, the membrane was not positioned perpendicular to the section plane and thus its visibility was affected by the anisotropic resolution of electron tomograms. We further analyzed only pairs of eisosome and plasma membrane that both yielded interpretable averages (Figures 3C and 3D). We confirmed the three-dimensional shapes of these eisosomes by manual segmentation of the membrane (Figure 3E).

We next compared the 2D class averages of the different eisosomes to those of the corresponding plasma membrane patches. The averages of eisosomes that were shallow appeared very similar to the averages of the nearby plasma membrane, displaying a single layer of density, likely representing the lipid bilayer (Figures 3C and 3D, I and II). The averages of furrow-shaped, curved eisosomes displayed two layers of density, while the averages of the corresponding plasma membrane displayed only one layer of density (Figures 3C and 3D, III–VI). The diameter of the outer density layer of the curved eisosomes ranged between 32 and 58 nm. This range is similar to the diameters reported for *in vitro* assembled Pil1 and Lsp1 lattices on liposomes (Karotki et al., 2011). To quantitatively assess the difference in appearance between shallow and curved eisosomes (Figure 3F), we measured the thickness of their membrane profiles and normalized it by the thickness of the nearby plasma membrane. The resulting relative membrane thickness of the shallow eisosomes was close to 1, indicating that they had the same thickness as the corresponding nearby plasma membranes (Figure 4A). Curved eisosomes were on average 1.6 times thicker than the corresponding plasma membrane. Furthermore, the relative membrane thickness of curved eisosomes differed significantly from that of shallow eisosomes (Figure 4A).

We conclude that curved eisosomal membranes were covered with a dense coat, which was neither present on the membrane of shallow eisosomes, nor on regions of the plasma membrane that did not correspond to eisosomes. Our data thus suggest that eisosomal proteins, including the BAR proteins Pil1 and Lsp1, are not arranged into a dense, regular protein coat when they are bound to low curvature membranes. The formation of a dense protein coat is associated with high membrane curvature, and the coat diameter varies between 30 and 60 nm (Figure 4B).

DISCUSSION

In this study, we implemented a cryo-correlative microscopy workflow for *in situ* visualization of elusive protein assemblies in thick regions of cells, with the goal of performing cellular structural biology studies. In particular, we surmounted two major bottlenecks that prevented routine *in situ* structural studies, namely efficient cryo-ET data acquisition from thick cellular samples and the unambiguous identification of structures within cells.

Imaging vitreous sections by cryo-FM allowed us to assess the attachment of sections to the EM grid and thereby dramatically increase the throughput of cryo-ET data acquisition. Although cryo-FM has been proposed to facilitate correlative microscopy of vitreous sections (Gruska et al., 2008), its benefits for cryo-ET have not been explored systematically. Poor attachment of sections to the EM grid can significantly impair the outcome of a cryo-ET session. It is possible to assess section attachment by tilting the grid (Al-Amoudi and Frangakis, 2013; Bouchet-Marquis and Hoenger, 2011). However, reloading grids, mapping them, tilting, and acquiring test images at multiple areas

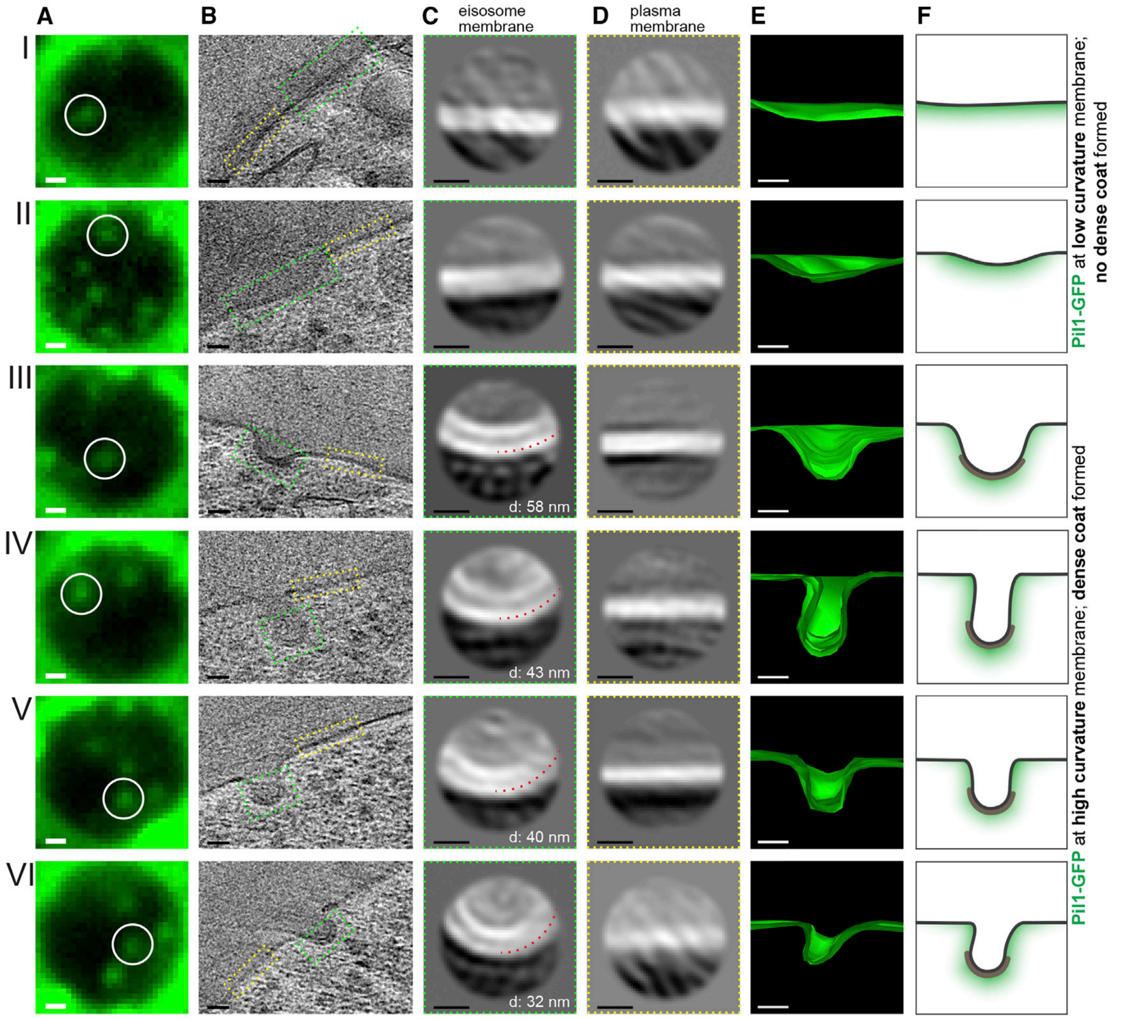

Figure 3. Sub-tomogram Averaging Analysis Reveals that Eisosomes with High Membrane Curvature Display a Dense Coat

(A) Cryo-FM (green channel) of section areas over carbon film holes containing Pil1-GFP signals of interest (white circles). Panel V corresponds to Figure 1E.

(B) Virtual slices through electron cryo-tomograms acquired at the corresponding positions.

(C) 2D class averages of eisosome membranes marked by green dashed boxes in (B). In III–VI, the diameter (d) of the outer density layer is indicated by the red dashed circle segment.

(D) 2D class averages of plasma membrane marked by yellow dashed boxes in (B).

(E) Corresponding 3D eisosome shape modeled by segmentation of the plasma membrane.

(F) Cartoon interpretation of coating and membrane curvature in presence of Pil1-GFP.

Scale bars represent 500 nm in (A), 25 nm in (B and E), and 10 nm in (C and D).

until good spots for acquisition are found are all time-consuming steps. This procedure may appear unfeasible on a 24-hr session as bookable in many national or institutional facilities. Therefore, we suggest that careful preselection of vitreous section areas by cryo-FM is an efficient means for assessing suitability for cryo-ET.

Combining cryo-ET of vitreous sections with a fiducial-based correlation procedure allowed us to unambiguously identify eisosomes in the yeast plasma membrane marked by the presence of Pil1-GFP. The diversity of eisosome shapes showed that the BAR protein Pil1 binds to membranes of widely differing curvature, including very shallow indentations. These shallow eisosomes would have been completely indiscernible without precise correlation to fluorescent signals, demonstrating the unique capability of our workflow to identify elusive cellular structures in cryo-EM.

The quality of the cryo-ET data allowed us to structurally analyze the protein assemblies on the cellular membranes identified as eisosomes. Sub-tomogram averaging revealed differences in protein organization on shallow and curved eisosome membranes. On eisosomes with high curvature, we observed a dense coat. This coat most likely corresponds to the regular lattice formed by a single layer of BAR proteins, as its thickness and diameter were remarkably consistent with the regular Pil1 lattice on lipid tubules (Karotki et al., 2011). A regular arrangement of proteins on curved eisosomes has also been suggested by EM of metal-shadowed cells and by the high Pil1 density measured in live cells (Karotki et al., 2011; Lacy et al., 2017).

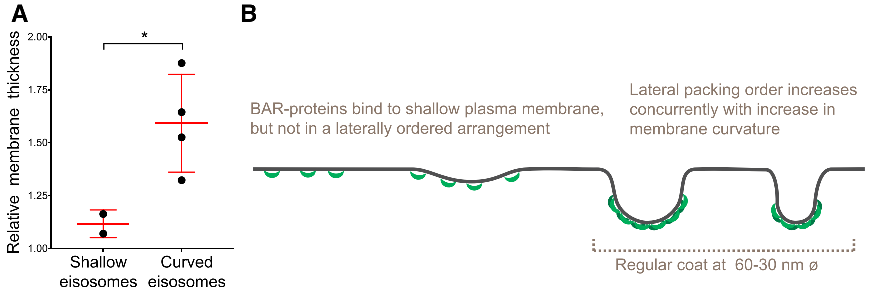

Figure 4. Eisosomal Protein Coating Correlates with Membrane Curvature

(A) Comparison of the relative membrane thickness of shallow (Figures 3I and 3II) and curved eisosomes (Figures 3III–3VI). *p = 0.0211. The red lines indicate the mean and the standard deviation.

(B) Model for the interdependence of BAR-domain scaffold formation and membrane curvature.

On shallow eisosome membranes, although labeled by Pil1-GFP similar to the curved ones, we did not observe such a dense protein coat. We conclude that while BAR proteins Pil1 and Lsp1 can bind to shallow membranes, they do not form a regular lattice.

Our finding that BAR proteins with a curved membrane-binding surface (Ziółkowska et al., 2011) bind to flat cellular membranes is in line with previous studies, which suggested that membrane binding and curvature induction by BAR domains can be uncoupled (Blood and Voth, 2006; Frost et al., 2008; Kelley et al., 2015). Furthermore, the propensity of BAR domains to induce curvature was proposed to depend on membrane tension and on the protein density on the membrane (Simunovic et al., 2015; Sorre et al., 2012). This model is supported by curved BAR protein assemblies that display dense or lattice-like coating of liposomes (Daum et al., 2016; Frost et al., 2008; Karotki et al., 2011; Mim et al., 2012). It has, however, been difficult to confirm this model *in vivo* because inside cells, flat membranes with low density of BAR proteins are difficult to identify. Our *in situ* data demonstrate that protein coating on shallow eisosomes is less dense than on curved ones, and that the formation of a dense BAR protein coat, likely in a regular, lattice-like organization, coincides with the formation of highly curved membrane furrows (Figure 4B).

Our data do not reveal whether the total number of BAR proteins bound to shallow eisosomes is lower than the number bound to curved eisosomes. The numbers of BAR protein molecules could be similar, but the molecules could be distributed over a different surface area, resulting in a different packing density. Shallow eisosomes could represent intermediates during their *de novo* formation in the buds of dividing yeast cells (Moreira et al., 2009). Pil1 patches are absent from small buds and appear during growth of the daughter cells (Moreira et al., 2009). Furrow-like invaginations are also absent from buds, but how Pil1 appearance and the formation of furrows are temporally coupled is unclear (Strádalová et al., 2009; Takeo, 1984). Alternatively, shallow eisosomes could represent an adaptation to increasing membrane tension (Kabeche et al., 2015).

In summary, we have implemented a workflow that permits localizing and structurally investigating elusive protein assemblies inside thick areas in the cell through efficient cryo-ET data acquisition. We demonstrate the potential of our approach by addressing the open question of how BAR-domain protein localization and arrangement correlates with membrane curvature in cells, difficult to answer by other means. Our workflow thereby complements the toolbox of cryo-correlative microscopy methods (Arnold et al., 2016; Hampton et al., 2017; Koning et al., 2014; Schellenberger et al., 2014; Schorb and Briggs, 2014) and could be applied to many other structural biology questions *in situ*.

STAR★METHODS

Detailed methods are provided in the online version of this paper and include the following:

- KEY RESOURCES TABLE
- CONTACT FOR REAGENT AND RESOURCE SHARING
- EXPERIMENTAL MODEL DETAILS
- METHOD DETAILS
  - Vitreous Sectioning
  - Cryo-FM of Vitreous Sections on EM Grids
  - Cryo-ET Data Collection
  - High-Precision Correlation Procedure
  - Sub-tomogram Analysis and Image Processing
  - Room Temperature Correlative Microscopy
- QUANTIFICATION AND STATISTICAL ANALYSIS
- DATA AVAILABILITY

SUPPLEMENTAL INFORMATION

Supplemental Information includes three figures and can be found with this article online at https://doi.org/10.1016/j.str.2018.03.015.

ACKNOWLEDGMENTS

T.A.M.B. is supported by a Sir Henry Dale Fellowship jointly funded by the Wellcome Trust and the Royal Society (grant no. 202231/Z/16/Z) and an EMBO advanced fellowship start-up grant (aALTF 778-2015). Work in W.K.'s group is supported by the Medical Research Council (MC_UP_1201/8). The authors would like to gratefully acknowledge helpful advice from Jan Löwe, advice on cryo-ET data collection from Wim Hagen, advice on statistics from Andrea Picco, and valuable comments on the manuscript from Harvey McMahon, Lori Passmore, Liz Miller, and Pedro Carvalho.

AUTHOR CONTRIBUTIONS

T.A.M.B and W.K. designed the experiments and wrote the paper with input from P.C.H. All authors conducted the research and contributed to data analysis.

DECLARATION OF INTERESTS

The authors declare no competing interests.

Received: December 14, 2017
Revised: February 22, 2018
Accepted: March 22, 2018
Published: April 19, 2018

REFERENCES

Ader, N.R., and Kukulski, W. (2017). triCLEM: Combining high-precision, room temperature CLEM with cryo-fluorescence microscopy to identify very rare events. Methods Cell Biol. *140*, 303–320.

Al-Amoudi, A., Chang, J.J., Leforestier, A., McDowall, A., Salamin, L.M., Norlén, L.P., Richter, K., Blanc, N.S., Studer, D., and Dubochet, J. (2004). Cryo-electron microscopy of vitreous sections. EMBO J. *23*, 3583–3588.

Al-Amoudi, A., Diez, D.C., Betts, M.J., and Frangakis, A.S. (2007). The molecular architecture of cadherins in native epidermal desmosomes. Nature *450*, 832–837.

Al-Amoudi, A., and Frangakis, A.S. (2013). Three-dimensional visualization of the molecular architecture of cell-cell junctions in situ by cryo-electron tomography of vitreous sections. Methods Mol. Biol. *961*, 97–117.

Arnold, J., Mahamid, J., Lucic, V., de Marco, A., Fernandez, J.J., Laugks, T., Mayer, T., Hyman, A.A., Baumeister, W., and Plitzko, J.M. (2016). Site-specific cryo-focused ion beam sample preparation guided by 3D correlative microscopy. Biophys. J. *110*, 860–869.

Asano, S., Fukuda, Y., Beck, F., Aufderheide, A., Förster, F., Danev, R., and Baumeister, W. (2015). Proteasomes. A molecular census of 26S proteasomes in intact neurons. Science *347*, 439–442.

Beck, M., and Baumeister, W. (2016). Cryo-electron tomography: can it reveal the molecular sociology of cells in atomic detail? Trends Cell Biol. *26*, 825–837.

Berchtold, D., Piccolis, M., Chiaruttini, N., Riezman, I., Riezman, H., Roux, A., Walther, T.C., and Loewith, R. (2012). Plasma membrane stress induces relocalization of Slm proteins and activation of TORC2 to promote sphingolipid synthesis. Nat. Cell Biol. *14*, 542–547.

Bharat, T.A., Russo, C.J., Löwe, J., Passmore, L.A., and Scheres, S.H. (2015). Advances in single-particle electron cryomicroscopy structure determination applied to sub-tomogram averaging. Structure *23*, 1743–1753.

Bharat, T.A., and Scheres, S.H. (2016). Resolving macromolecular structures from electron cryo-tomography data using subtomogram averaging in RELION. Nat. Protoc. *11*, 2054–2065.

Blood, P.D., and Voth, G.A. (2006). Direct observation of Bin/amphiphysin/Rvs (BAR) domain-induced membrane curvature by means of molecular dynamics simulations. Proc. Natl. Acad. Sci. USA *103*, 15068–15072.

Bouchet-Marquis, C., and Hoenger, A. (2011). Cryo-electron tomography on vitrified sections: a critical analysis of benefits and limitations for structural cell biology. Micron *42*, 152–162.

Daum, B., Auerswald, A., Gruber, T., Hause, G., Balbach, J., Kühlbrandt, W., and Meister, A. (2016). Supramolecular organization of the human N-BAR domain in shaping the sarcolemma membrane. J. Struct. Biol. *194*, 375–382.

Fröhlich, F., Christiano, R., Olson, D.K., Alcazar-Roman, A., DeCamilli, P., and Walther, T.C. (2014). A role for eisosomes in maintenance of plasma membrane phosphoinositide levels. Mol. Biol. Cell *25*, 2797–2806.

Frost, A., Perera, R., Roux, A., Spasov, K., Destaing, O., Egelman, E.H., De Camilli, P., and Unger, V.M. (2008). Structural basis of membrane invagination by F-BAR domains. Cell *132*, 807–817.

Gallop, J.L., Jao, C.C., Kent, H.M., Butler, P.J.G., Evans, P.R., Langen, R., and McMahon, H.T. (2006). Mechanism of endophilin N-BAR domain-mediated membrane curvature. EMBO J. *25*, 2898–2910.

Gruska, M., Medalia, O., Baumeister, W., and Leis, A. (2008). Electron tomography of vitreous sections from cultured mammalian cells. J. Struct. Biol. *161*, 384–392.

Hagen, W.J., Wan, W., and Briggs, J.A. (2017). Implementation of a cryo-electron tomography tilt-scheme optimized for high resolution subtomogram averaging. J. Struct. Biol. *197*, 191–198.

Hampton, C.M., Strauss, J.D., Ke, Z., Dillard, R.S., Hammonds, J.E., Alonas, E., Desai, T.M., Marin, M., Storms, R.E., Leon, F., et al. (2017). Correlated fluorescence microscopy and cryo-electron tomography of virus-infected or transfected mammalian cells. Nat. Protoc. *12*, 150–167.

Hohmann-Marriott, M.F., Sousa, A.A., Azari, A.A., Glushakova, S., Zhang, G., Zimmerberg, J., and Leapman, R.D. (2009). Nanoscale 3D cellular imaging by axial scanning transmission electron tomography. Nat. Methods *6*, 729–731.

Hsieh, C.E., Leith, A., Mannella, C.A., Frank, J., and Marko, M. (2006). Towards high-resolution three-dimensional imaging of native mammalian tissue: electron tomography of frozen-hydrated rat liver sections. J. Struct. Biol. *153*, 1–13.

Iudin, A., Korir, P.K., Salavert-Torres, J., Kleywegt, G.J., and Patwardhan, A. (2016). EMPIAR: a public archive for raw electron microscopy image data. Nat. Methods *13*, 387–388.

Kabeche, R., Howard, L., and Moseley, J.B. (2015). Eisosomes provide membrane reservoirs for rapid expansion of the yeast plasma membrane. J. Cell Sci. *128*, 4057–4062.

Karotki, L., Huiskonen, J.T., Stefan, C.J., Ziółkowska, N.E., Roth, R., Surma, M.A., Krogan, N.J., Emr, S.D., Heuser, J., Grünewald, K., and Walther, T.C. (2011). Eisosome proteins assemble into a membrane scaffold. J. Cell Biol. *195*, 889–902.

Kelley, C.F., Messelaar, E.M., Eskin, T.L., Wang, S., Song, K., Vishnia, K., Becalska, A.N., Shupliakov, O., Hagan, M.F., Danino, D., et al. (2015). Membrane charge directs the outcome of F-BAR domain lipid binding and autoregulation. Cell Rep. *13*, 2597–2609.

Koning, R.I., Celler, K., Willemse, J., Bos, E., van Wezel, G.P., and Koster, A.J. (2014). Correlative cryo-fluorescence light microscopy and cryo-electron tomography of *Streptomyces*. Methods Cell Biol. *124*, 217–239.

Kremer, J.R., Mastronarde, D.N., and McIntosh, J.R. (1996). Computer visualization of three-dimensional image data using IMOD. J. Struct. Biol. *116*, 71–76.

Kukulski, W., Schorb, M., Kaksonen, M., and Briggs, J.A. (2012). Plasma membrane reshaping during endocytosis is revealed by time-resolved electron tomography. Cell *150*, 508–520.

Kukulski, W., Schorb, M., Welsch, S., Picco, A., Kaksonen, M., and Briggs, J.A. (2011). Correlated fluorescence and 3D electron microscopy with high sensitivity and spatial precision. J. Cell Biol. *192*, 111–119.

Lacy, M.M., Baddeley, D., and Berro, J. (2017). Single-molecule imaging of the BAR domain protein Pil1p reveals filament-end dynamics. Mol. Biol. Cell *28*, 2251–2259.

Marko, M., Hsieh, C., Schalek, R., Frank, J., and Mannella, C. (2007). Focused-ion-beam thinning of frozen-hydrated biological specimens for cryo-electron microscopy. Nat. Methods *4*, 215–217.

Mastronarde, D.N. (2005). Automated electron microscope tomography using robust prediction of specimen movements. J. Struct. Biol. *152*, 36–51.

McDonald, K. (2007). Cryopreparation methods for electron microscopy of selected model systems. Methods Cell Biol. *79*, 23–56.

McMahon, H.T., and Gallop, J.L. (2005). Membrane curvature and mechanisms of dynamic cell membrane remodelling. Nature *438*, 590–596.

Mim, C., Cui, H., Gawronski-Salerno, J.A., Frost, A., Lyman, E., Voth, G.A., and Unger, V.M. (2012). Structural basis of membrane bending by the N-BAR protein endophilin. Cell *149*, 137–145.

Mindell, J.A., and Grigorieff, N. (2003). Accurate determination of local defocus and specimen tilt in electron microscopy. J. Struct. Biol. *142*, 334–347.

Moreira, K.E., Walther, T.C., Aguilar, P.S., and Walter, P. (2009). Pil1 controls eisosome biogenesis. Mol. Biol. Cell *20*, 809–818.

Olivera-Couto, A., Salzman, V., Mailhos, M., Digman, M.A., Gratton, E., and Aguilar, P.S. (2015). Eisosomes are dynamic plasma membrane domains showing pil1-lsp1 heteroligomer binding equilibrium. Biophys. J. *108*, 1633–1644.

Peter, B.J., Kent, H.M., Mills, I.G., Vallis, Y., Butler, P.J.G., Evans, P.R., and McMahon, H.T. (2004). BAR domains as sensors of membrane curvature: the amphiphysin BAR structure. Science *303*, 495–499.

Pettersen, E.F., Goddard, T.D., Huang, C.C., Couch, G.S., Greenblatt, D.M., Meng, E.C., and Ferrin, T.E. (2004). UCSF Chimera–a visualization system for exploratory research and analysis. J. Comput. Chem. *25*, 1605–1612.

Pfeffer, S., Woellhaf, M.W., Herrmann, J.M., and Förster, F. (2015). Organization of the mitochondrial translation machinery studied in situ by cryoelectron tomography. Nat. Commun. *6*, 6019.

Pierson, J., Fernandez, J.J., Bos, E., Amini, S., Gnaegi, H., Vos, M., Bel, B., Adolfsen, F., Carrascosa, J.L., and Peters, P.J. (2010). Improving the technique of vitreous cryo-sectioning for cryo-electron tomography: electrostatic charging for section attachment and implementation of an anti-contamination glove box. J. Struct. Biol. *169*, 219–225.

Sartori Blanc, N., Studer, D., Ruhl, K., and Dubochet, J. (1998). Electron beam-induced changes in vitreous sections of biological samples. J. Microsc. *192*, 194–201.

Schellenberger, P., Kaufmann, R., Siebert, C.A., Hagen, C., Wodrich, H., and Grünewald, K. (2014). High-precision correlative fluorescence and electron cryo microscopy using two independent alignment markers. Ultramicroscopy *143*, 41–51.

Scheres, S.H. (2012). RELION: implementation of a Bayesian approach to cryo-EM structure determination. J. Struct. Biol. *180*, 519–530.

Schorb, M., and Briggs, J.A. (2014). Correlated cryo-fluorescence and cryo-electron microscopy with high spatial precision and improved sensitivity. Ultramicroscopy *143*, 24–32.

Schorb, M., Gaechter, L., Avinoam, O., Sieckmann, F., Clarke, M., Bebeacua, C., Bykov, Y.S., Sonnen, A.F., Lihl, R., and Briggs, J.A. (2016). New hardware and workflows for semi-automated correlative cryo-fluorescence and cryo-electron microscopy/tomography. J. Struct. Biol. *197*, 83–93.

Seger, S., Rischatsch, R., and Philippsen, P. (2011). Formation and stability of eisosomes in the filamentous fungus *Ashbya gossypii*. J. Cell Sci. *124*, 1629–1634.

Simunovic, M., Voth, G.A., Callan-Jones, A., and Bassereau, P. (2015). When physics takes over: BAR proteins and membrane curvature. Trends Cell Biol. *25*, 780–792.

Sorre, B., Callan-Jones, A., Manzi, J., Goud, B., Prost, J., Bassereau, P., and Roux, A. (2012). Nature of curvature coupling of amphiphysin with membranes depends on its bound density. Proc. Natl. Acad. Sci. USA *109*, 173–178.

Strádalová, V., Stahlschmidt, W., Grossmann, G., Blaziková, M., Rachel, R., Tanner, W., and Malinsky, J. (2009). Furrow-like invaginations of the yeast plasma membrane correspond to membrane compartment of Can1. J. Cell Sci. *122*, 2887–2894.

Takeo, K. (1984). Lack of invaginations of the plasma membrane during budding and cell division of *Saccharomyces cerevisiae* and *Schizosaccharomyces pombe*. FEMS Microbiol. Lett. *22*, 97–100.

Walther, T.C., Brickner, J.H., Aguilar, P.S., Bernales, S., Pantoja, C., and Walter, P. (2006). Eisosomes mark static sites of endocytosis. Nature *439*, 998–1003.

Youn, J.Y., Friesen, H., Kishimoto, T., Henne, W.M., Kurat, C.F., Ye, W., Ceccarelli, D.F., Sicheri, F., Kohlwein, S.D., McMahon, H.T., et al. (2010). Dissecting BAR domain function in the yeast amphiphysins Rvs161 and Rvs167 during endocytosis. Mol. Biol. Cell *21*, 3054–3069.

Ziółkowska, N.E., Karotki, L., Rehman, M., Huiskonen, J.T., and Walther, T.C. (2011). Eisosome-driven plasma membrane organization is mediated by BAR domains. Nat. Struct. Mol. Biol. *18*, 854–856.

STAR★METHODS

KEY RESOURCES TABLE

REAGENT or RESOURCE	SOURCE	IDENTIFIER
Chemicals, Peptides, and Recombinant Proteins		
TetraSpeck Microspheres 100 nm	Invitrogen / Thermo Fisher Scientific	Catalog number T7279
TetraSpeck Microspheres 50 nm	Invitrogen / Thermo Fisher Scientific	Custom order
Dextran from Leuconostoc spp. Mr ~40000	Sigma	Product 68084
Lowicryl HM20 embedding kit	Polysciences, Inc.	Catalog number 15924
Deposited Data		
Electron cryo-tomogram of vitreous section of yeast cell	This paper, deposited at EMDB	EMD-4305
Electron cryo-tomographic tilt series and corresponding correlative microscopy data	This paper, deposited at EMPIAR	EMPIAR-10161
Experimental Models: Organisms/Strains		
S. cerevisiae strain: *MATα, his3Δ200, leu2-3,112, ura3-52, lys2-801, PIL1-EGFP::HIS3MX6*	This paper	WKY0207
Software and Algorithms		
RELION	Bharat et al., 2015<br>Scheres, 2012	http://www2.mrc-lmb.cam.ac.uk/relion
MATLAB-based correlation scripts	Kukulski et al., 2011<br>Schorb et al., 2016	NA
SerialEM	Mastronarde 2005	http://bio3d.colorado.edu/SerialEM/
IMOD	Kremer et al., 1996	http://bio3d.colorado.edu/imod/
CTFFIND	Mindell and Grigorieff, 2003	NA
Other		
QUANTIFOIL EM grids (copper, 200 mesh, R3.5/1)	www.quantifoil.com	No product code
Gold-coated copper specimen carrier type A	Wohlwend GmbH, Sennwald, Switzerland	Art. 662
Aluminium specimen carrier type B	Wohlwend GmbH, Sennwald, Switzerland	Art. 242
Aluminium specimen carrier type A	Wohlwend GmbH, Sennwald, Switzerland	Art. 241
Carbon film EM grids (copper, 200 mesh)	Agar Scientific	Code AGS160

CONTACT FOR REAGENT AND RESOURCE SHARING

Further information and requests for resources and reagents should be directed to and will be fulfilled by the Lead Contact, Wanda Kukulski (kukulski@mrc-lmb.cam.ac.uk).

EXPERIMENTAL MODEL DETAILS

Saccharomyces cerevisiae expressing Pil1-GFP (genotype: *MATα, his3Δ200, leu2-3,112, ura3-52, lys2-801, PIL1-EGFP::HIS3MX6)* were grown at 30°C in SC-Trp medium to exponential phase (OD_{600} ~0.8) and pelleted by vacuum filtration (McDonald, 2007) for high pressure freezing.

METHOD DETAILS

Vitreous Sectioning

Pelleted yeast paste was transferred into the 0.2 mm recess of gold-coated copper carrier type A (Wohlwend GmbH) by pipetting, mixed to a ratio of ~1:1 with a solution of 10 nm colloidal gold beads (BBI solutions) in 40% Dextran (Molecular mass ~40,000 Da, Sigma), covered with the flat side of an aluminium carrier type B and high pressure frozen using a Leica HPM100. Vitreous sectioning

was conducted in an EM UC6/FC6 cryo-microtome (Leica Microsystems), using cryotrim 20 and 35° cryo immuno diamond knives (Diatome), at 1 mm/s speed and 150 nm feed. 100 nm TetraSpeck beads (Invitrogen), diluted 1:50 in phosphate buffer saline were adhered to plasma cleaned Quantifoil R 3.5/1 on Cu 200 mesh grids (Quantifoil) by incubation for approximately 5 minutes, blotting and subsequent washing with three drops of water. Vitreous sections were attached using the Crion antistatic device (Leica Microsystems) to grids held in the FC6 cryo-chamber by a micromanipulator. The grids were transferred to cryo-grid boxes and stored in liquid nitrogen until cryo-FM imaging.

Cryo-FM of Vitreous Sections on EM Grids

Fluorescence imaging of vitreous sections was performed on the Leica EM cryo-CLEM system with a HCX PL APO 50x cryo-objective with NA = 0.9 (Leica Microsystems), an Orca Flash 4.0 V2 sCMOS camera (Hamamatsu Photonics), a Sola Light Engine (Lumencor) and the following filters: L5 excitation 480/40, dichroic 505, emission 527/30 and N21 excitation 515-560, dichroic 580, emission LP 590 (Leica Microsystems). During imaging, the humidity of the room was controlled to 20-25% and the microscope stage was cooled to -195°C. At first, using the Leica LAS X software, a 7x7 overview tile scan around the center of the grid was acquired in bright field (BF) and green channel with an autofocus range in the z-direction of 80 μm (4 μm step size) in BF. Short exposure times and low light intensities (BF channel: 10 ms exposure, intensity 30; green channel: 1 s exposure, intensity 17%) were used to limit potential radiation damage to the sample. This overview tile scan was used to identify the sections and grid orientation in cryo-EM. Individual ribbons of vitreous sections on the grid were subsequently imaged in the BF, green and red channel over a focal range of approximately 6 to 10 μm, set to obtain focused images of all section areas, with a step size of 0.3 μm. The following channel settings were used: 50 ms exposure, intensity 30 in BF channel; 3 s exposure, 30% intensity in green channel; 1.5 s exposure, 30% intensity in red channel. Using these conditions, grids were imaged up to 120 minutes each without noticeable increase in contamination or de-vitrification of the sections. Grid squares with attached sections, which displayed GFP signals and auto-fluorescence of the carbon film in the same focal plane, were preselected using the ImageJ software. The overview tile scan and the focal stacks of vitreous sections were then used for visual correlation during cryo-ET data acquisition. Cryo-fluorescence images shown in the figures have been adjusted for contrast individually, thus absolute intensities from different figure panels are not directly comparable.

Cryo-ET Data Collection

Cryo-ET data collection was performed on a Titan Krios microscope (FEI) fitted with a Quantum energy filter (slit width 20 eV) and a K2 direct electron detector (Gatan) running in counting mode with a dose rate of ~ 8 e^-/pixel/second at the detector level during tilt series acquisition. Montaged images of the entire grid were acquired at low magnification (pixel size 191 nm) to localize grid squares preselected by cryo-FM. Intermediate magnification maps of these grid squares were acquired at a pixel size of 5.1 nm. Using these intermediate magnification maps, all those positions that showed a high degree of compression, contamination or a possible rupture of the plasma membrane at its interface with the cell wall were discarded. In the intermediate magnification maps, we visually predicted locations of the preselected Pil1-GFP signals. Since the frame size of the electron cryo-tomograms was approximately 1.3 μm, the accuracy of the prediction did not need to be very high to ensure that the eisosome would be contained within the tomogram. We therefore found that for positioning cryo-ET data acquisition, performing a computational prediction of the signal based on transformation algorithms at this stage was not strictly necessary. Tilt series of the cryo-FM correlated eisosome sites were collected between ±60° using a grouped dose-symmetric tilt scheme (Hagen et al., 2017) implemented in SerialEM (Mastronarde, 2005). Rather than acquiring single images in opposite directions, groups of four tilted images with a 1° tilt increment were collected successively before tilting the goniometer to the other side of the 0° tilt angle (Figure S1). A total dose of 110 $e^-/Å^2$ was applied over the entire series, and data was sampled at a calibrated pixel size of 3.5 Å.

High-Precision Correlation Procedure

Correlation between the cryo-FM and cryo-EM images was conducted using custom MATLAB-based scripts as described previously (Kukulski et al., 2011; Schorb and Briggs, 2014). For each correlation (see also Figure S2), TetraSpeck beads (fiducial markers) were localized in a single frame from the montaged intermediate magnification images that contained the cell of interest. In one case (Figure S2VII), a blended montage of single frames (IMOD) was required to localize enough fiducials around the cell of interest for correlation. Pil1-GFP signal and TetraSpeck signals were picked in a single green channel image selected from the focal stacks.

Sub-tomogram Analysis and Image Processing

CTF estimation of each image in each tilt series was performed in CTFFIND (Mindell and Grigorieff, 2003). CTF correction of the tilt series and tomographic reconstructions using patch tracking were performed in IMOD (Kremer et al., 1996). Membrane profiles along each eisosome were clicked in Z-slices through the eisosomal site periodically at 15 nm intervals. Similarly, profiles of the membrane roughly 200 nm away from each eisosome were also clicked. A spline was fitted through each set of clicked points (MATLAB) and sub-tomograms were extracted along the fitted spline in (40%) overlapping boxes in the RELION software (Bharat et al., 2015). The in-plane rotation angle from the spline fit was also written out and retained for subsequent alignments. Each sub-tomogram was collapsed onto a 2D image (Bharat and Scheres, 2016), and subjected to two-dimensional averaging using a regularized-likelihood algorithm (Scheres, 2012). For each membrane site, multiple classifications were performed with different number of output class averages to control for different curvatures of the eisosomal membrane. Selection of well-aligned classes was performed by careful visual inspection of results from different runs. Data visualization was performed in IMOD and UCSF Chimera (Pettersen et al., 2004).

IMOD was also used for segmentation of eisosome membranes, which was performed on tomographic reconstructions binned to a pixel size of 7 Å. Coat curvature in the 2D class averages was estimated by applying the function imodcurvature in IMOD to a set of points clicked along the densest area of the coat. For better visibility, the virtual slices shown in the figures are also from tomographic reconstructions binned to a pixel size of 7 Å, and have been subjected to mild Gaussian filtering.

Room Temperature Correlative Microscopy

Room-temperature correlative microscopy was conducted as described in (Kukulski et al., 2011), with minor modifications described in (Ader and Kukulski, 2017). Briefly, pelleted yeast paste was high-pressure frozen in Aluminium carriers (Wohlwend) using a HPM100 (Leica Microsystems). Freeze-substitution and Lowicryl HM20 (Polysciences, Inc.) embedding followed the protocol described in (Kukulski et al., 2011), except that the uranyl acetate concentration during freeze-substitution was 0.03%. 300 nm sections on 200 mesh carbon-coated copper grids (AGS160, Agar Scientific), incubated with 50 nm TetraSpeck beads (Invitrogen) were imaged on a TE2000-E microscope (Nikon) using a 100x TIRF objective, a NEO sCMOS DC-152Q-C00-FI camera (Andor), using filter sets 49002 ET GFP (Chroma), excitation 470/40, dichroic T495LP, emission 525/50 for GFP and 49006 ET CY5 (Chroma), excitation 520/60, dichroic T660lpxr, emission 700/75 for far-red signal (Ader and Kukulski, 2017). Scanning transmission EM tomography was performed on a TF20 microscope (FEI), equipped with an axial bright-field detector, using a camera length of 200 mm and a C2 aperture of 50 μm (Ader and Kukulski, 2017; Hohmann-Marriott et al., 2009). Tilt series of ±60° tilt range were acquired using Serial EM (Mastronarde, 2005), at a pixel size of 3.1 nm, 2° increment as single axis (for correlation to FM images), and a pixel size of 1.1 nm, 1° increment, as dual axis. Tomographic reconstructions were performed using IMOD (Kremer et al., 1996). Correlation was done using MATLAB-based scripts as described in (Kukulski et al., 2011).

QUANTIFICATION AND STATISTICAL ANALYSIS

To estimate the thickness of the membranes including a potential protein coat, average line profiles of the central 7 pixel lines for each of the class averages shown in Figure 3 were generated. The line profiles showed a major single or double peak, corresponding to the membrane and coat densities, and a dip on the side of the major peak, corresponding to the extracellular side of the membrane. The mean gray scale value between the gray scale value of the highest peak and the gray scale value of the extracellular dip was calculated. At the mean gray scale value, the width of the major peak was measured, which was used together with the calibrated pixel size as an estimate for thickness of the membrane including a potential protein coat. In this way, membrane thickness values for the eisosomes as well as the corresponding plasma membrane patches were obtained. To calculate the "relative membrane thickness" shown in Figure 4A, the thickness of the eisosome membrane was divided by the thickness of the corresponding plasma membrane patch (less than ~200 nm away from the eisosome). The relative membrane thickness of shallow eisosomes (shown in Figures 3I and 3II) was compared to that of curved eisosomes (shown in Figures 3III–3VI) using a two-tailed Welch test, assuming that the data is normally distributed (significance shown in Figure 4A).

DATA AVAILABILITY

The cryo-ET tilt series and corresponding correlative microscopy data have been deposited in EMPIAR (Iudin et al., 2016) under accession code EMPIAR-10161. A representative cryo-ET reconstruction (corresponding to data shown in Figures 3VI and S2VI) has been deposited in the EMBD under accession code EMD-4305.

Structure, Volume *26*

Supplemental Information

Correlative Microscopy of Vitreous Sections Provides Insights into BAR-Domain Organization *In Situ*

Tanmay A.M. Bharat, Patrick C. Hoffmann, and Wanda Kukulski

Supplemental Figure S1

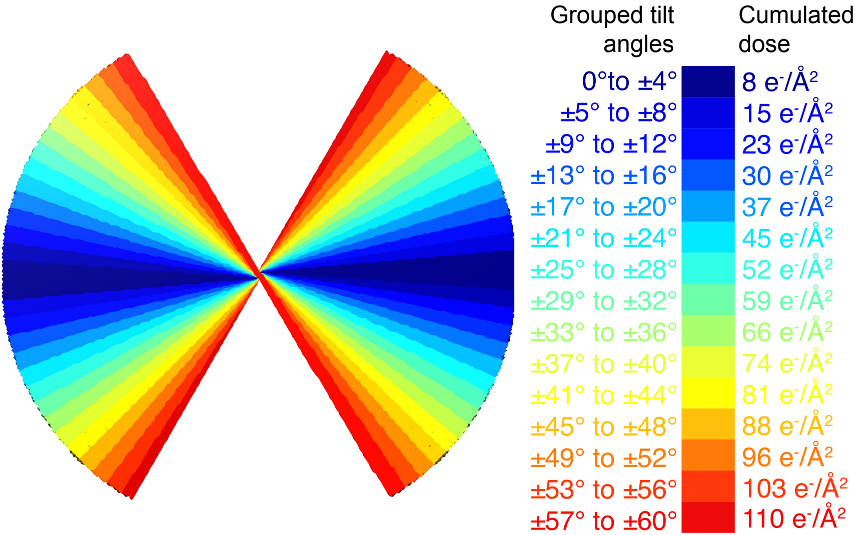

Supplemental Figure S2

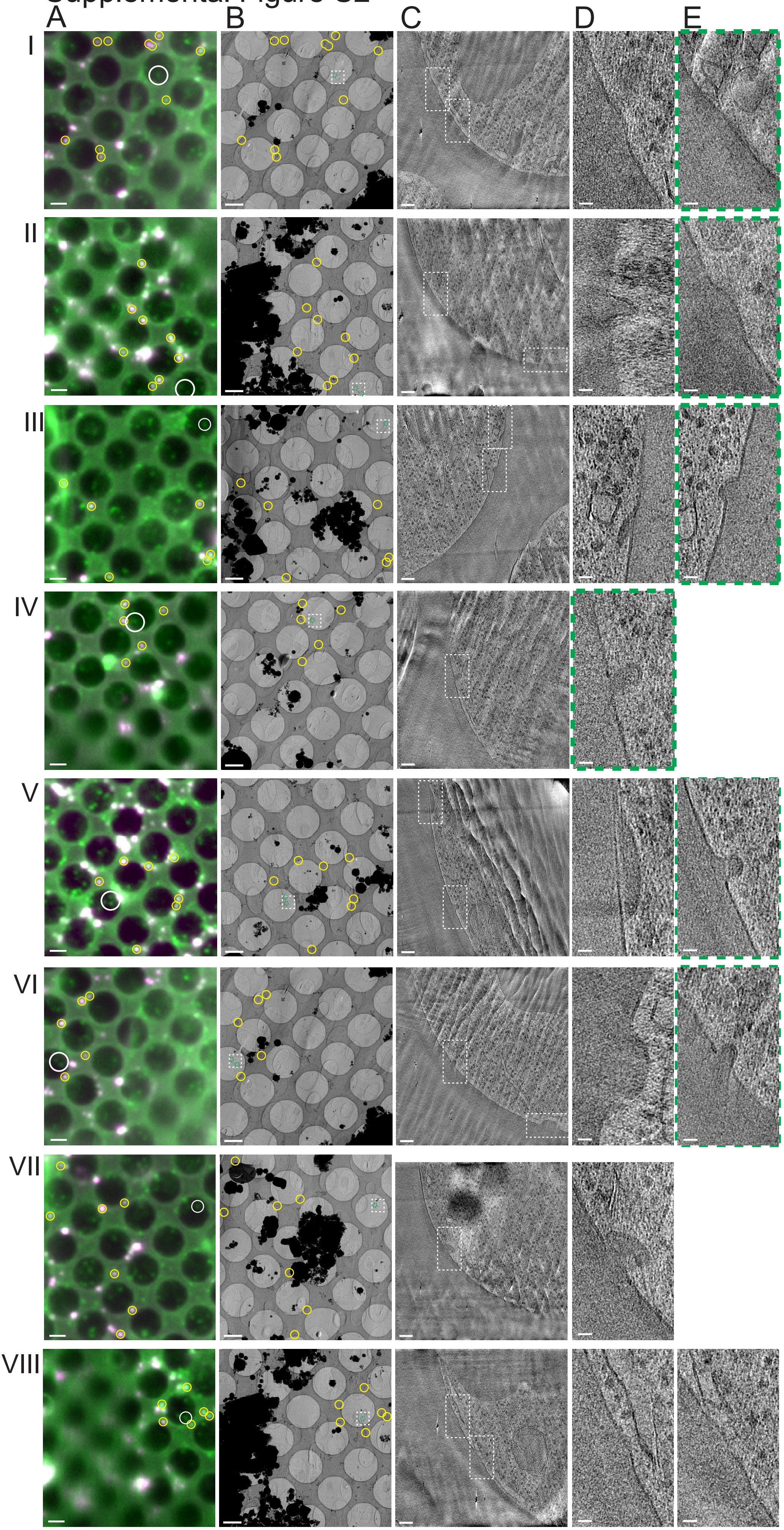

Supplemental Figure S3

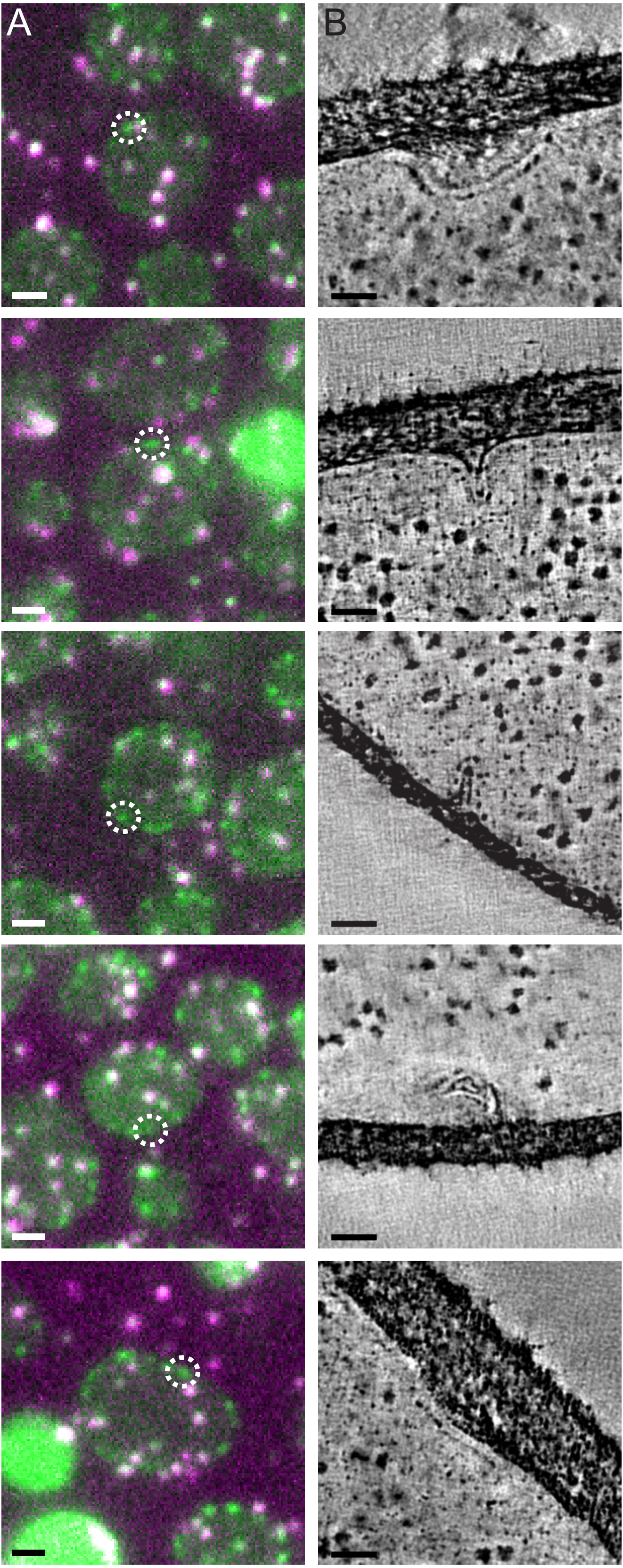

Supplemental Figure Legends:

Supplemental Figure S1: Tilt scheme. Related to Figure 2. We applied a grouped dose-symmetric tilt scheme for cryo-ET data acquisition, where the goniometer is successively tilted in opposite directions (Hagen et al., 2017). Four images were collected in succession in one direction (relative to the untilted 0° image), followed by four images in the other direction, while moving from 0° to ±60°. Changing direction after every four images proved to be time-efficient, as well as dose-efficient, since high cumulated doses were restricted to high tilt images. This allowed discarding high tilt images without compromising the lower tilt images.

Supplemental Figure S2: Gallery of cryo-correlative microscopy and corresponding cryo-ET data. Related to Figures 2 and 3. A: Single focal plane cryo-FM images, merge of red (shown in magenta) and green channels, rotated, cropped and scaled to match the corresponding intermediate magnification cryo-EM images shown in **B**. The coordinates of the Pil1-GFP signals indicated by the white circle (A), were transformed using the fiducial markers highlighted by the yellow circles in A and B. The centres of the green dashed circles (B) are the predicted positions of Pil1-GFP signals of interest. Note that the white circles (A) in rows II, V and VI contain two Pil1-GFP signals each. The white dashed rectangles correspond to the field of view imaged by cryo-ET, shown in **C** as a single virtual slice through the reconstructed tomogram. Dashed rectangles in C indicate the location of the eisosomes shown in **D** and **E**. The images in D and E are magnifications of the virtual slice shown in C, except for EI and EII, which are magnifications of different virtual slices than those shown in C. Green dashed boxes indicate eisosomes that are also shown in Figure 3 and that have resulted in interpretable 2D class averages of eisosomal as well as nearby plasma membrane. Due to a defect of the direct electron detector, the EM images have a stripe of faulty pixels. They appear as a narrow stripe of repeating image features apparent in some of the images shown in B. Images shown in

EI and Figure 3BI, EII and Figure 3BII, DIV and Figure 3BIV, and EV and Figure 3BV, are details from the same image, respectively. Scale bars: 2 *μ*m in A and B, 100 nm in C and 25 nm in D and E.

Supplemental Figure S3: Room-temperature correlative light and electron microscopy of Pil1-GFP. Related to Figure 3. Room-temperature correlative light and electron microscopy confirms that eisosomes correspond to furrow-like invaginations that are oriented at various angles relative to the plane of the plasma membrane, as well as shallow indentations of the plasma membrane. **A:** Room-temperature fluorescence microscopy of 300 nm sections of yeast cells embedded in resin, on EM grids. Overlay of green (Pil1-GFP) and far-red (fiducial markers, shown in magenta) signals. **B:** Virtual slices through scanning transmission electron microscopy tomograms acquired at the predicted positions of the Pil1-GFP spots marked by white dashed circles in A. Scale bars: 1 *μ*m in A, 50 nm in B.
